#  Empowering Without Misinforming Adolescents and Young Adults with Cystic Fibrosis. Comment on “Perceptions of Social Media Use to Augment Health Care Among Adolescents and Young Adults With Cystic Fibrosis: Survey Study” 

**DOI:** 10.2196/33457

**Published:** 2022-05-25

**Authors:** Navandeep Thumber, Prerana Bhandari

**Affiliations:** 1 St Georges University of London Medical School London United Kingdom

**Keywords:** cystic fibrosis, social media, mobile health, adherence, adolescents, young adults, medical misinformation

We would like to thank the authors of the original research published in JMIR Pediatrics and Parenting [1] for exploring the attitudes toward social media and the capacity in which it can serve to enhance health care delivery for patients with cystic fibrosis (CF). Despite how far we have come in our understanding of the disease and the significant improvement in the care and overall prognosis of this cohort of patients, studies have shown that adolescents and young adults with CF continue to face significant barriers in their care concerning increasing CF complications, issues with medication compliance, and notably increased isolation and mental health vulnerabilities [1]. This article highlights thoughtful avenues in which social media may play a role in addressing some of these issues, such as access to medical information and education and the creation of online patient forums for peer social support. As the COVID-19 pandemic has forced us away from traditional face-to-face practice of medicine and further toward the realm of telemedicine and the digital world, this article is even more pertinent in its notes on the interplay between social media and health care today.

However, the pandemic has also exposed the uncapped danger that social media poses in the form of medical misinformation. One aspect that this article could have considered is the prevalence of medical misinformation and how this may limit the role of social media in aiding health care services for patients with CF.

The spread of false medical information has become a public health crisis in recent years [2]. A study examining the spread of misinformation of the Zika virus showed misinformation was three times more likely to be shared on social media than verified stories [3]. This study found that, of the 50 adolescents and young adults with CF who participated in the survey study and reviewed CF-related information online, 55% rarely or never checked to ensure the source of the medical content they consumed was accurate [1]. Not only does misinformation dilute factual content [4], it also has the potential to severely impact a patient’s quality of life and risk of mortality [3]. Vosoughi et al [5] hypothesized that false information spreads faster than facts because users identify more with its content, as it often elicits a strong emotional response. Considering patients with CF are a much younger demographic, it should be considered whether these patients are more vulnerable to misinformation.

If we, as health care professionals, advocate social media to our patients, we should also consider our role in ensuring they are being exposed to the correct information and sources. However, the practicalities of this can be argued: how can we ensure patients view only credible sources or whether individuals can evaluate the validity of such sources? Moving forward, further research must be conducted to ensure how we can, practically and safely, implement social media guidelines for these patients, to empower adolescents and young adults with CF and provide them with a safe space to access information related to their condition.

## Abbreviations

CF: cystic fibrosis.
